# Differences between the real and the desired worlds in the results of clinical trials

**DOI:** 10.6061/clinics/2015(09)04

**Published:** 2015-09

**Authors:** Regina El Dib, Eliane Chaves Jorge, Amélia Kamegasawa, Solange Ramires Daher, Regina Stella Spagnuolo, Marise Pereira da Silva, Gabriel Pereira Braga, Enilze Volpato, Norma Sueli Pinheiro Módolo, Marluci Betini, Adriana do Valle, Ione Corrêa, Rodrigo Bazan, Ricardo Augusto MB Almeida, Silke Anna Theresa Weber, Silvana Molina, Hugo Yoo, Paulo Villas Boas, José Eduardo Corrente, Joseph Mathew, Anil Kapoor, Raíssa Pierri Carvalho, Roberto Bezerra Vital, Leandro Gobbo Braz, Paulo do Nascimento Junior

**Affiliations:** I Universidade Estadual Paulista (Unesp), Faculdade de Medicina, Botucatu/SP, Brazil; II McMaster University, McMaster Institute of Urology, Canada/USACanada/USA; III Universidade Estadual Paulista (Unesp), Bioscience Institute, Biostatistics Department, Botucatu/SP, Brazil; IV Pediatric Pulmonology, PGIMER, Chandigarh, India; V McMaster University, McMaster Institute of Urology, Division of Urology Program Director, Urology Residency Program Surgical Director, Canada/USACanada/USA

**Keywords:** Clinical Medicine, Clinical Trials, Evidence-Based Medicine, Research, Efficacy, Effectiveness

## Abstract

**OBJECTIVE:**

We refer to the effectiveness (known as pragmatic or real world) and efficacy (known as explanatory or desired or ideal world) of interventions. However, these terms seem to be randomly chosen by investigators who design clinical trials and do not always reflect the true purpose of the study. A pragmatic-explanatory continuum indicator summary tool was thus developed with the aim of identifying the characteristics of clinical trials that distinguish between effectiveness and efficacy issues. We verified whether clinical trials used the criteria proposed by the indicator summary tool, and we categorized these clinical trials according to a new classification.

**METHOD:**

A systematic survey of randomized clinical trials was performed. We added a score ranging from 0 (more efficacious) to 10 (more effective) to each domain of the indicator summary tool and proposed the following classifications: high efficacy (<25), moderate efficacy (25-50), moderate effectiveness (51-75), and high effectiveness (<75).

**RESULTS:**

A total of 844 randomized trials were analyzed. No analyzed trials used the criteria proposed by the indicator summary tool. Approximately 44% of the trials were classified as having moderate effectiveness, and 43.82% were classified as having moderate efficacy.

**CONCLUSIONS:**

Most clinical trials used the term “efficacy” to illustrate the application of results in clinical practice, but the majority of those were classified as having moderate effectiveness according to our proposed score. The classification based on the 0-100 score is still highly subjective and can be easily misunderstood in all domains based on each investigator's own experiences and knowledge.

## INTRODUCTION

Clinical trials produce the best data available for health care decision making, as they use a prospective design, follow a process of randomization (patients are allocated at random to receive one of several clinical interventions) and seek to measure and compare the outcomes of two or more clinical treatments. The randomized clinical trial (RCT) in particular is one of the most simple, powerful and revolutionary tools in research 1.

When discussing treatment and examining evidence, we refer to effectiveness (known as pragmatic or management related; treatment that works under real-world conditions) and efficacy (known as explanatory; treatment that works under ideal conditions) 2,3. However, these terms seem to be randomly chosen by investigators who design clinical trials and do not always reflect the true purpose of the study. In addition, renowned educational institutions involved in evidence-based medicine disseminate vague definitions of these terms 2,3, as described in 1967 by Schwartz 4.

A pragmatic-explanatory continuum indicator summary (PRECIS) tool 5 was developed in 2009 with the aim of identifying the characteristics of clinical trials that distinguish between effectiveness and efficacy issues and assisting researchers in preparing their clinical trials. It is important to note that according to PRECIS 5, the classification of a clinical trial is not a dichotomy, i.e., there is a gradient between effectiveness and efficacy. Therefore, it is very difficult to conduct (and hence categorize) a clinical trial as “purely” one of effectiveness or “purely” one of efficacy.

However, it is unclear whether investigators designing clinical trials use PRECIS 5 to assist policy makers and health professionals with trials' results in policy and clinical practice. Additionally, it is unclear whether the PRECIS tool would be adequate to classify trials into real- or desired-world categories.

Therefore, we i) verified whether clinical trials published in the last three years used the criteria proposed by PRECIS 5; ii) determined whether the clinical trials' authors made appropriate use of the terms “effectiveness” and “efficacy” according to a proposed 0-100 scale, called the Grading of Efficacy-Effectiveness in Clinical Trials (GEECT); and iii) classified the clinical trials according to the GEECT classification (i.e., high or moderate efficacy, high or moderate effectiveness).

## METHODS

### Overall study design

In this systematic survey, we randomly selected full reports of randomized clinical trials (RCTs) published in journals that were chosen based on their importance to clinical practice. Table 1 shows the list of journals analyzed and their respective percentages, which were considered in our clinical trial search strategy.

### Eligibility criteria

The inclusion criteria were as follows:

- RCTs published in full-text in one of the selected journals (Table 1) and
- RCTs mentioning the terms “effectiveness” and/or “efficacy” in either the title or the objectives section.

### The Grading of Efficacy-Effectiveness in Clinical Trials (GEECT) tool

We added a score ranging from 0 (more efficacious) to 10 (more effective) to each of the 10 domains of the PRECIS 5 tool (i.e., eligibility criteria for trial participants, flexibility with which the experimental intervention is applied, degree of practitioner expertise in applying the experimental intervention, flexibility with which the comparison intervention is applied, degree of practitioner expertise in applying the comparison intervention, follow-up of trial participants, trial's primary outcome, participants' compliance with the prescribed intervention, practitioners' adherence to the study protocol, and analysis of the primary outcome), which together determine the extent to which a trial is effective or efficacious, and we named it the GEECT tool.

We then created a classification, with definitions ranging from high efficacy (HEcy) to high effectiveness (HEss), as shown in Table 2. The terms “effectiveness” and “efficacy” were defined according to the PRECIS 5 tool.

The agreement variable “Is the term used correctly?” was related to the concordance between the terms “efficacy” and/or “effectiveness” used by an RCT's authors and the GEECT classification, as ranked by the investigators of the present study using the GEECT score. For example, if an RCT's authors used the term “efficacy” and the GEECT classification fell into either the HEcy or moderate efficacy (MEcy) category, we answered “yes, the term was used correctly” for this question. In contrast, if an RCT's authors used the term “efficacy” and the GEECT classification fell into either the HEss or moderate effectiveness (MEss) category, we answered “no, the term was not used correctly” for this question. Finally, if an RCT's authors used both “efficacy” and “effectiveness,” we considered the use of both terms in their published papers.

### Selection of studies

We searched studies published between 2009 and 2012 in each chosen journal using a comprehensive search strategy, including an exhaustive list of synonyms for clinical trials and the words “effectiveness” and “efficacy.” This search was specifically conducted by a librarian.

### Data extraction

Phase 1 consisted of brainstorming and conducting practical exercises with all of the investigators involved in the data extraction via weekly meetings over eight months. Phase 2 comprised a pilot study, with all of the investigators scoring the same RCT according to the GEECT classification.

### Statistical analysis

We randomized 47 clinical trials for consistency checking by two randomly chosen investigators and calculated the inter-observer agreement rate using the kappa test in relation to the GEECT classification. We expressed each domain as the mean and standard deviation (SD). The overall mean and SD from each investigator's data were used. The percentage of the GEECT classification (i.e., HEcy, MEcy, MEss and HEss) was also calculated. Proportional tests were conducted to compare MEcy and MEss as well as HEcy and HEss. The results were considered significant when p was <0.05. We used SPSS software version 12.0.

## RESULTS

A total of 1,039 references were identified. From this total, 844 RCTs met the inclusion criteria and were analyzed by 19 investigators from different areas of expertise. Therefore, each investigator analyzed approximately 44 clinical trials in duplicate and independently. The remaining studies were generally excluded because the terms “effectiveness” and/or “efficacy” were found in either the discussion or the reference section.

The studies were published in a wide variety of journals (33 journals), with 14.34% in the New England Journal of Medicine, followed by 9.83% in The Lancet and 8.89% in the Journal Cancer (Table 1).

The inter-observer agreement for the GEECT categories classified as HEcy, MEcy, MEss and HEss was slight (kappa coefficient: 0.11) for the two investigators.

No clinical trials published in the selected journals in the last three years used the criteria proposed by PRECIS to differentiate between effectiveness and efficacy.

The statistics relating to the 10 domains, ranging from 0 (more efficacious/ideal/desired) to 10 (more effective/real), that were found in the RCTs and analyzed by the 19 investigators are shown below as the mean (SD):

4.98 (1.47) - all participants who have the condition of interest are enrolled, regardless of their anticipated risk, responsiveness, comorbidities or past compliance;

4.08 (1.45) - instructions on how to apply the experimental intervention are highly flexible, offering practitioners considerable leeway in deciding how to formulate and apply it.

5.50 (1.82) - the experimental intervention is typically applied by the full range of practitioners and in the full range of clinical settings, regardless of expertise, with only ordinary attention to dose setting and side effects.

4.14 (1.30) - “usual practice” or the best alternative management strategy available is recommended, offering practitioners considerable leeway in deciding how to apply it.

5.77 (2.06) - the comparison intervention is typically applied by the full range of practitioners and in the full range of clinical settings, regardless of expertise, with only ordinary attention to practitioner training, experience and performance.

4.53 (1.85) - no formal follow-up visits with study individuals are performed. Instead, administrative databases (e.g., mortality registries) are searched for the detection of outcomes.

5.70 (2.00) - the primary outcome is an objectively measured, clinically meaningful outcome for the study participants. This outcome does not rely on central adjudication and is one that can be assessed under usual conditions (e.g., special tests and training are not required).

5.44 (1.98) - there is unobtrusive (or no) measurement of participant compliance. No special strategies to maintain or improve compliance are used.

5.30 (2.34) - there is unobtrusive (or no) measurement of practitioner adherence. No special strategies to maintain or improve practitioner adherence to the study protocol are used.

6.38 (1.82) - the analysis includes all patients, regardless of compliance, eligibility, and other factors (such as intention-to-treat analysis). In other words, the analysis attempts to determine if the treatment works under the usual conditions, with all of the noise inherent therein.

The overall mean and SD for the GEECT score throughout the 844 clinical trials were 53.20 and 12.16, respectively.

**Figure 1:**
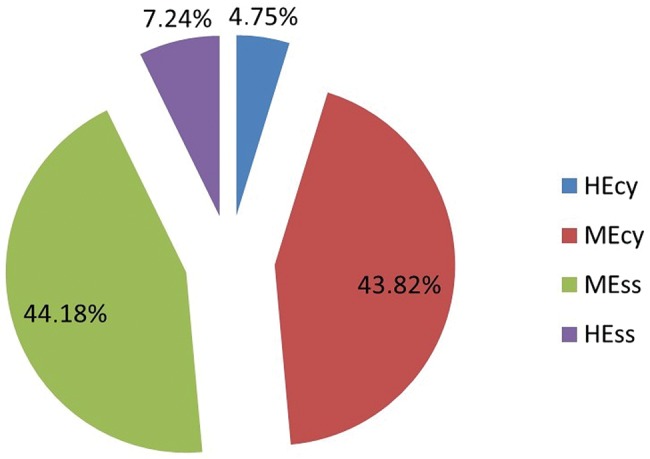
The Grading of Efficacy-Effectiveness in Clinical Trials (GEECT) classification throughout the 844 clinical trials analyzed.

The percentages of GEECT classifications are shown in Figure 1. Approximately 44% of the trials were classified as MEss, followed by 43.82% as MEcy. There were no statistically significant differences between MEcy and MEss or between HEcy and HEss (*p*=0.921 and *p*=0.052, respectively).

A total of 735 clinical trials used the term “efficacy,” and 392 RCTs used the term “effectiveness.” Moreover, 301 clinical trials used both “efficacy” and “effectiveness” in their published papers. In 359 clinical trials, the clinical trial authors made appropriate use of the terms “effectiveness” and “efficacy” according to the ranking by our investigators using the proposed GEECT score. In contrast, in 192 trials, there was a discrepancy between the term chosen by the authors and the investigators' proposed GEECT score.

## DISCUSSION

Clinical trials are indeed the best primary study design to answer questions about treatment and prevention. Due to their essential importance in clinical practice, there is an urgent need to distinguish between effectiveness and efficacy so that health professionals and policy makers can adequately use the findings of trials in their respective settings.

The first tool to evaluate whether an RCT's results would work under ideal or real conditions was developed by Thorpe and colleagues 5. After that tool was developed, further studies were published with the aim of determining the extent of the usefulness of the PRECIS tool, as one of the major problems encountered with the tool developed by Thorpe and colleagues was its highly subjective approach.

In the present study, the highest mean value (6.38) presented throughout the 844 trials analyzed was for the domain “the analysis includes all patients regardless of compliance, eligibility, and other factors,” suggesting a gradient with a tendency toward effectiveness. Meanwhile, the lowest mean value (4.08) was related to the domain “instructions on how to apply the experimental intervention are highly flexible,” which offers practitioners considerable leeway in deciding how to formulate and apply an intervention.

Overall, the trials were classified between MEss and MEcy, which indicates that few trials are “purely” effective or “purely” efficacious and confirms the existence of a gradient between the real and the desired worlds in the results of clinical trials. 

Our kappa value showed the difficulties in grading any RCT with regard to a desired- or real-world scenario. Even adding a score ranging from 0 (more efficacious/ideal/desired) to 10 (more effective/real) to each of the 10 domains of the PRECIS 5 tool, such as eligibility criteria, follow-up, and adherence to the study protocol, did not make the new proposed tool more objective. In fact, it is still very difficult to rate each domain of a clinical trial and to try to distinguish between real- and desired-world results.

One study 6 described the use of the PRECIS tool in helping to resolve debates within a trial team on whether an ongoing clinical trial of two drug treatments for a rare blistering skin disease was more pragmatic or more explanatory. The authors concluded that the PRECIS tool can be used to retrospectively determine pragmatism, and they provided several recommendations for using this tool.

Another study 7 applied the PRECIS criteria to a set of trials and reported experience with a rating system scored on a 0-4 scale. The authors emphasized the need for more comprehensive reporting on PRECIS and indicated that the criteria proposed by PRECIS' collaborators may not be sufficient to provide a precise profile of a clinical trial's applications.

In 2011, Koppenaal et al. 8 modified the PRECIS tool (generating the PRECIS-Review tool) to grade individual trials and systematic reviews of trials. The trials included in two systematic reviews were specifically scored on the 10 PRECIS domains on a scale of 1-5. The authors concluded that the modified PRECIS tool with a 1-5 scale is less subjective for researchers and policy makers.

Furthermore, Riddle 9 and colleagues described the usefulness of the PRECIS tool to facilitate discussion and decisions regarding the pragmatic-explanatory continuum-related issues arising from a clinical trial's results. The researchers found that the PRECIS tool was useful in aiding discussions related to trial design, revisions to clinical trial design and achievement of consensus.

Additionally, a 2012 study 10 added a 20-point numerical rating scale to the PRECIS tool to assist in the design of a particular trial focused on smoking cessation. After discussion, the authors concluded that there was consensus on all 10 domains of the study design, and the study scored high on pragmatism.

Although we have proposed a quantitative score in an attempt to reduce subjectivity, we feel that this is still not sufficient, as there might be a tendency to grade toward the central value if investigators are unclear on how to proceed with the score. Furthermore, the clinical expertise of the investigator seems to make a difference in how each domain of the modified PRECIS tool is ranked, as indicated by the finding of slight agreement in the kappa test in the current study. Another issue is the lack of information reported in the RCTs examined in the present study, which made it even more difficult to rank them from 0-10.

Moreover, when an RCT reported on both intention-to-treat and per-protocol analyses, we gave it a score of five, but this method introduced bias into the final GEECT score and most likely affected the RCT's classification.

Even though we encountered these issues, RCTs' authors should be encouraged to explore these tools when designing their studies, and policy makers can use the existing tools while further instruments to reduce the subjectivity of such tools are under investigation.

Loudon et al. 11 proposed a study to improve and validate a version of the PRECIS tool and to compare the internal validities of a set of explanatory and pragmatic trials matched by intervention.

Furthermore, there is another prominent approach to this problem, which is termed “pragmatic-mechanistic” 12,13. In particular, authors have proposed a new mechanistic-practical framework for designing and interpreting RCTs because they believe that there are major limitations (such as confusing purpose with structure) to the interpretation of the explanatory-pragmatic framework. However, several authors do not agree with how these arguments were framed, but they recognize that the mechanists have identified an important problem: how pragmatic a trial is depends on one's perspective and context 14.

The GEECT classification based on the 0-100 score is still highly subjective and can be easily misunderstood in all of the domains based on each investigator's own experiences, knowledge, and values. However, we found the following with respect to the clinical trials in the literature over the last three years:

All of the clinical trials published in the last three years that we analyzed did not use the criteria proposed by the PRECIS tool to differentiate between the real and the desired worlds.

The majority of the clinical trials' authors made appropriate use of the terms “effectiveness” and “efficacy” according to the rankings by our investigators using the GEECT scale, but the relatively common use of both terms (effectiveness and efficacy) in the same publication makes it difficult to use the scalein the development of health policies.

- The majority of the trials studied were classified as MEss/real world (51-75), followed by MEcy/desired world (25-50), according to the GEECT tool.
- Most clinical trials published in the analyzed journals in the last three years used the term “efficacy” to illustrate the application of results in clinical practice.

More research is needed to establish the easiest and most useful tool to a) facilitate the applicability of results in clinical practice, b) distinguish between effectiveness (real world) and efficacy (desired world) results and c) assist researchers in preparing and planning clinical trials.

We also suggest that after the establishment of a more appropriate and objective tool to determine the specific application of a clinical trial's results (i.e., more effective or more efficacious), journals all over the world that publish clinical trials should request the submission of a quantitative score related to effectiveness or efficacy by authors, along with their full research articles. Furthermore, journals should publish a note with regard to the effectiveness and efficacy scores that accompany the main text of a clinical trial.

## Figures and Tables

**Table 1:** List of the journals analyzed and their respective percentages.

Journal's name	Number (%)
New England Journal of Medicine	121 (14.34)
The Lancet	83 (9.83)
Cancer	75 (8.89)
JAMA: The Journal of the American Medical Association	55 (6.52)
The Clinical Journal of Pain	51 (6.04)
Ophthalmology	44 (5.21)
Archives of Internal Medicine	32 (3.79)
Clinical Infectious Diseases	31 (3.67)
Circulation	29 (3.44)
The Journal of Pediatrics	28 (3.32)
British Journal of Anaesthesia	27 (3.20)
American Journal of Respiratory and Critical Care Medicine	26 (3.08)
European Urology	26 (3.08)
Anesthesiology	21 (2.49)
European Journal of Heart Failure	21 (2.49)
American Journal of Ophthalmology	20 (2.37)
Transplantation	20 (2.37)
Annals of Surgery	15 (1.78)
The European Respiratory Journal	13 (1.54)
Thorax	13 (1.54)
Annals of Neurology	10 (1.18)
Hypertension	9 (1.07)
The American Journal of Medicine	9 (1.07)
BMJ: British Medical Journal	6 (0.71)
Archives of Surgery	6 (0.71)
Journal of Clinical Epidemiology	6 (0.71)
American Journal of Surgery	5 (0.59)
Critical Care Medicine	5 (0.59)
Occupational and Environmental Medicine	5 (0.59)
The American Journal of Surgery	5 (0.59)
Diabetes	4 (0.47)
Journal of Epidemiology and Community Health	4 (0.47)
Science	4 (0.47)
Journal of Internal Medicine	3 (0.36)
Kidney International	3 (0.36)
American College of Physicians	2 (0.24)
Annals of Internal Medicine	2 (0.24)

**Table 2:** Classification using the Grading of Efficacy-Effectiveness in Clinical Trials (GEECT) tool.

Classification (abbreviation) (score range)	Definition
**High Efficacy (HEcy) (<25)**	Research is very likely to apply under ideal conditions.
**Moderate Efficacy (MEcy) (25-50)**	Research is likely to apply under ideal conditions, but certain variables are more flexible than others (e.g., the primary outcome is an objectively measured, clinically meaningful outcome for the study participants).
**Moderate Effectiveness (MEss) (51-75)**	Research is likely to apply under real conditions, but certain variables are stricter than others (e.g., study individuals are followed via many, more frequent visits and more extensive data collection than would occur in routine practice).
**High Effectiveness (HEss) (<75)**	Research is very likely to apply under real conditions.
